# Phenotypic and genotypic analysis of pediatric nephronophthisis patients with different levels of proteinuria

**DOI:** 10.1080/0886022X.2025.2598179

**Published:** 2025-12-15

**Authors:** Qiuxia Chen, Jing Ouyang, Fei Zhao, Hongmei Wu, Quancheng Feng, Bixia Zheng, Chunhua Zhu

**Affiliations:** ^a^ Department of Nephrology, Children’s Hospital of Nanjing Medical University, Nanjing, China; ^b^ Nanjing Key Laboratory of Pediatrics, Children’s Hospital of Nanjing Medical University, Nanjing, China

**Keywords:** Nephronophthisis, children, ESRD, proteinuria, NPHP1, TTC21B

## Abstract

While nephronophthisis (NPHP) classically manifests as mild tubular proteinuria, emerging evidence reports nephrotic-range proteinuria with edema. This study aims to explore the phenotypes and genotypes of pediatric NPHP patients with different levels of proteinuria. Twenty-one children with NPHP were recruited into this single-center cohort. They were divided into two groups according to proteinuria levels: group A (mild proteinuria, *n* = 12) and group B (moderate-to-heavy proteinuria, *n* = 9). The median age at symptom onset was 7.4 years (IQR 2.4–12.8). By the end of follow-up, 20 patients had progressed to ESRD at a median age of 9.8 years (IQR 5.8–12.9). Patients in group B exhibited significantly higher prevalence of family histories, hypertension, edema, and higher urine microalbumin-to-α1-microglobulin (MA/A1M) ratios. Genetic testing identified *NPHP1* mutations as the most prevalent (61.9%), followed by *TTC21B* variants (19.0%). However, *TTC21B* mutations were more commonly found in group B (3/4, 75.0%) with one homozygous mutation and two compound heterozygous mutations. Five novel mutations of NPHP genes were identified. Further literature review of patients with *TTC21B* gene mutations revealed that the P209L variant was almost present in all North African/European patients, while the C518R heterozygous mutation occurred in 72.7% of Asian patients. In conclusion, NPHP remains a diagnosis in patients presenting with moderate-to-heavy proteinuria, particularly when accompanied by characteristic features including positive family history, hypertension, and edema, with *TTC21B* emerging as the predominant pathogenic gene in this clinical subgroup.

## Introduction

Nephronophthisis (NPHP), a rare autosomal recessive cystic kidney disease with an incidence of 1:50,000 to 1:1,000,000 live births [1], manifests as either isolated nephropathy or multisystem involvement and represents the leading genetic cause of end-stage renal disease (ESRD) in patients under 30 years [2,3]. According to the age of progression to ESRD, NPHP is classified into three clinical groups: infantile, juvenile, and adolescent/adult, with median ESRD onset ages of 1, 13 and 19 years, respectively. Juvenile NPHP is the most prevalent form.

NPHP primarily affects cilia, which are classified as motile or immotile (primary) types, with the latter being ubiquitously expressed on renal tubular epithelial cells. While most NPHP patients demonstrate an insidious onset characteristic of tubulointerstitial diseases (polyuria, polydipsia, secondary enuresis, or anemia), only a minority present with overt edema, oliguria, or hypertension [4,5], leading to frequent misdiagnosis or diagnostic delays in clinical practice.

The functions of distinct ciliary domains are tightly linked to specific genes, with over 90 causative genes currently identified for NPHP-related ciliopathies (NPHP-RC) [6]. *NPHP1* remains the most prevalent (52.8% incidence [7]), followed by *TTC21B* variants (6% in Petzold’s cohort), which are also established as pathogenic for familial focal segmental glomerular sclerosis (FSGS) [8,9] and the patients may present with edema, nephrotic-range proteinuria and hypertension. Notably, other NPHP-associated genes can similarly manifest with significant proteinuria [10]. It is worth mentioning that the same causative gene sometimes cause significantly different degrees of proteinuria [9,11]. Therefore, is there any correlation between the specific gene mutation and the severity of proteinuria as well as other clinical phenotypes?

This study presents the first systematic characterization of genotype-phenotype correlations in pediatric NPHP patients stratified by proteinuria severity. A single-center NPHP cohort was established and divided into two groups (mild proteinuria vs. moderate-to-heavy proteinuria). The clinical data and genetic results were analyzed, identifying five novel mutations. *TTC21B* mutations were more prevalent in the moderate-to-heavy proteinuria group. Literature review indicates that most *TTC21B* patients presenting with moderate-to-heavy proteinuria carry homozygous or compound heterozygous variants. P209L and C518R are hotspot mutations in North African and Chinese populations, respectively.

## Methods

### Study design and participants

The data of children diagnosed with NPHP were analyzed in this study. The inclusion criteria included: (1) visited our center from January 1, 2018 to December 31, 2024; (2) exhibiting at least one clinical manifestation of NPHP, including polyuria, polydipsia, growth retardation, anemia, and low-molecular-weight proteinuria; (3) genetic confirmation of NPHP-associated gene mutations by whole exome sequencing (WES); (4) availability of complete clinical data; and (5) age ≤18 years at diagnosis. Exclusion criteria comprised: (1) cases lacking genetic confirmation; (2) patients with incomplete clinical data; and (3) individuals with comorbid primary/secondary renal pathologies (e.g. diabetic nephropathy, lupus nephritis).

First-morning void samples (collected on three consecutive days) during their initial visit to our center were analyzed after excluding infected/contaminated specimens. Protein-to-creatinine ratio (UPCR) was quantified using Roche assays (TPUC3 for protein; CREJ2 for creatinine). The patients were divided into two groups based on the level of proteinuria: group A (mild proteinuria), defined as urine dipstick < 2+ or UPCR < 1 g/g; group B (moderate-to-heavy proteinuria), characterized by urine dipstick ≥ 2+ or UPCR ≥ 1 g/g.

The study was approved by the Ethics Committee of Children’s Hospital of Nanjing Medical University (202503055-1). Written informed consent was obtained from patients or their parents prior to their enrollment in the study.

### Data collection

Clinical data were collected from medical records, including gender, physical examinations, age and symptoms at onset, age at ESRD diagnosis (defined as eGFR <15 mL/min/1.73m^2^ per KDIGO guidelines [12]), and family history. Laboratory data primarily consisted of urinalysis parameters and urinary protein component analysis, including urinary microalbumin (MA), immunoglobulin G (IgG), and the low-molecular-weight marker α-1 microglobulin (A1M). All patients received abdominal ultrasound, electrocardiogram, and chest X-ray during their first visit to our center. Patients with neurological abnormalities detected by physical examination underwent cranial magnetic resonance imaging (MRI). Renal biopsy was performed in patients with acute disease progression and heavy proteinuria. All patients were followed up regularly and effectively through outpatient clinic and telephone consultations.

### Genetic analyses

WES and bioinformatic analysis were conducted by the Beijing Chigene Institute. Genomic DNA was extracted from the peripheral blood of patient/parent, using TIANGEN Blood Genome Magnetic Genomic DNA Kit (DP329-TA). Qualified DNA then underwent exome capture with xGen Exome Research Panel v2.0 (IDT, Iowa, USA). The enrichment libraries were sequenced on MGI DNBSEQ-T7 platforms (≥99% target coverage at 30× depth). Raw data processing included adapter/low-quality read removal *via* BWA alignment to GRCh37/hg19, with variant calling (single nucleotide polymorphisms, SNPs; insertions/deletions, Indels) performed using Genome Analysis Toolkit (GATK) (depth/quality-filtered) and annotation against OMIM, the Human Genome Mutation Database (HGMD), ClinVar, gnomAD, dbSNP, 1000 Genomes, ESP, ExAC, and Chigene’s MAF database. The potential impact of variants on protein structure and function was predicted by Provean, Sift, Polyphen-2, Mutationtaster, and M-Cap. Variant interpretation was performed by bioinformaticians, clinical molecular geneticists and nephrologists, with final classifications (“pathogenic”/”likely pathogenic”) determined per ACMG/AMP guidelines through integrated genotype-phenotype correlation analysis, while variants of uncertain significance (VUS) in known disease-associated genes were also retained for clinical consideration. Pathogenic and likely pathogenic variants were validated by Sanger sequencing and subjected to parental segregation analysis.

### Statistical analyses

All statistical analyses in this study were performed using SPSS V.27. Age was expressed as either median (interquartile range [IQR]) or mean ± standard deviation (SD). Body mass index (BMI) and UPCR were reported as mean ± SD. Continuous variables were compared using the unpaired t-test or Mann-Whitney *U* test, and categorical variables with Fisher’s exact test. *p* < 0.05 was considered statistically significant.

## Results

### Demographic data and phenotype

This single-center cohort enrolled 21 pediatric NPHP patients (M: F = 10:11). There were 12 cases (P1-P12) in group A (7 males, 58.3%) and 9 cases (p1–p9) in group B (3 males, 33.3%), the latter showing greater familial aggregation (56% vs. group A, *p* < 0.05) with sibling mortality (p1/p6), familial ESRD (p3/p4), and consanguinity (p2) (Table 1).

**Table 1.** The demographic data and phenotype of two groups.

Parameters	Group A (*n* = 12)	Group B (*n* = 9)	Total (*n* = 21)	*p*
Gender/male, *n* (%)	7 (58.3)	3 (33.3)	10 (47.6)	0.387
Family history, *n* (%)	1 (8.3)	5 (55.6)	6 (28.6)	**0.046**
Age at onset (yr), median (IQR)	8.1 (5.1–10.3)	6.3 (1.3–13.3)	7.4 (2.4–12.8)	0.696
With ESRD, *n* (%)	11 (91.7)	9 (100)	20 (95.2)	>0.999
Age at ESRD (yr), median (IQR)	9.8 (8.4–11.6)^a^	6.3 (2.8–13.3)	9.8 (5.8–12.9)^b^	0.518
Infantile NPHP, *n* (%)	1 (9.1)^a^	3 (33.3)	4 (20.0)^b^	0.285
Juvenile NPHP, *n* (%)	10 (90.9)^a^	5 (55.6)	15 (75.0)^b^	0.127
HBP, *n* (%)	0 (0)	5 (55.6)	5 (23.8)	**0.006**
Edema, *n* (%)	0 (0)	4 (44.4)	4 (19.1)	**0.021**
BMI, mean ± SD	16.1 ± 3.1	16.6 ± 4.4^c^	16.2 ± 3.5^d^	0.768

Note: ESRD: End-stage renal disease; NPHP: Nephronophthisis; HBP: High blood pressure; BMI: Body mass index; Data presented as count, median (interquartile range, IQR), or mean ± standard deviation (SD); One patient in group A was currently in CKD4, so ^a^*n* = 11, ^b^*n* = 20; The BMI index of two patients in group B was not found, so ^c^*n* = 7, ^d^*n* = 19; Bold indicates *p* < 0.05.

The median age of disease onset was 7.4 years (IQR 2.4–12.8), with 8.1 years (IQR 5.1–10.3) of group A and 6.3 years (IQR 1.3–13.3) of group B (Table 1). Clinical presentations included anemia/fatigue (*n* = 13, 61.9%), proteinuria (*n* = 2), elevated serum creatinine (*n* = 2), gastrointestinal symptoms (*n* = 2), edema (*n* = 1), and growth retardation (*n* = 1). The median age at the first clinic visit was 9.6 years (IQR 4.3–12.8), with no intergroup disparity [group A: 9.7 years (7.0–11.3); group B: 6.3 years (1.8–13.3)]. Initial evaluation showed polyuria in 9.5% (2/21) of group A compared to oliguria in 14.3% (3/21) of group B. Hypertension (23.8%, 5/21) and associated edema (in 80% of hypertensive cases) were found exclusively in group B. Growth assessment identified 15.8% (3/19) with significant short stature per Chinese pediatric height standards (2023), and mean BMI was 16.2 ± 3.5 kg/m^2^ (Table 1).

At initial presentation, 71.4% of patients (15/21, group A:8, group B:7) had progressed to ESRD, with all but one (patient P8 maintaining CKD stage 4 throughout follow-up) ultimately reaching ESRD (95.2% total), comprising 4 infantile NPHP (20.0%), 15 juvenile NPHP (75.0%) and 1 adolescent NPHP. The median age of progression to ESRD was 9.8 years (IQR 5.8–12.9), with 9.8 years (IQR 8.4–11.6) of group A and 6.3 years (IQR 2.8–13.3) of group B. As shown in Table 1, no statistically significant differences were observed between the groups in ESRD incidence, age at ESRD onset, or the distribution of infantile NPHP and juvenile NPHP subtypes. Extrarenal manifestations were absent in 16 patients (76.2%). Intractable hemolytic anemia, thrombocytopenia, as well as multiple xanthomas were simultaneously showed by patient P9 due to the merger of sitosterolemia. P10 was diagnosed with Joubert syndrome, characterized by congenital cerebral palsy and the presence of a “molar tooth sign” on MRI. One group B patient (p2) exhibited situs inversus (Supplementary Table 1).

### Laboratory and imaging examination

A significant intergroup difference in proteinuria was observed, with group A demonstrating lower mean UPCR (0.6 ± 0.2 g/g) compared to group B (2.8 ± 1.6 g/g, *p* < 0.001), paralleled by distinct urinary MA/A1M ratios [group A: 0.3 (0.2–0.7) vs. group B: 4.1 (1.7–13.3), *p* < 0.001]. No hematuria cases occurred, while mild glycosuria (28.6%, 6/21) and reduced urinary specific gravity (14.3%, 3/21) were noted. Renal ultrasonography per Kadioglu A’s criteria [13] revealed normal/reduced kidney size in 85.7% (18/21), with enlargement in 3 cases (group A:2, group B:1) and multiple cysts in 28.6% (6/21) (Supplementary Table 2). Renal biopsies from five patients (group A:3; group B:2) comprised *NPHP1* (*n* = 2), *TTC21B* (*n* = 2), and *NPHP3* (*n* = 1) genotypes. The sole FSGS case (patient p1 with *TTC21B* mutation) progressed to ESRD within four years despite multimodal therapy, while others exhibited characteristic tubulointerstitial fibrosis with ischemic changes.

### Genetic analysis results

WES was performed for all children, revealing *NPHP1* as the most prevalent causative gene (61.9%), exclusively through homozygous large deletions with higher occurrence in group A (75.0%) versus group B (44.4%), while *TTC21B* variants (second most frequent) were identified in four patients (group B:3/4) (Table 2, Supplementary Table 2). The verification of patients with compound heterozygous mutations by Sanger sequencing is presented in Supplementary Figure 1. Through database searches up to January 15, 2015, there were five novel variants identified in our study: two *NPHP2* heterozygous variants (group B), along with three heterozygous mutations (*NPHP3* and *TTC21B* in group A; *CCDC41* in group B).

**Table 2.** The laboratory examination and genotype of two groups.

Parameters	Group A (*n* = 12)	Group B (*n* = 9)	Total (*n* = 21)	*p*
UPCR (g/g), mean ± SD	0.6 ± 0.2	2.8 ± 1.6	1.6 ± 1.5	**<0.001**
Urinary MA /A1M, median (IQR)	0.3 (0.2–0.7)	4.1 (1.7–13.3)	0.8 (0.2–3.6)	**<0.001**
Low urine S.G., *n* (%)	3 (25.0)	0 (0)	3 (14.3)	0.229
Renal cysts by ultrasound, *n* (%)	3 (25.0)	3 (33.3)	6 (28.6)	>0.999
*NPHP1* mutation, *n* (%)	9 (75.0)	4 (44.4)	13 (61.9)	0.203
*TTC21B* mutation, *n* (%)	1 (8.3)	3 (33.3)	4 (19.0)	0.272

Note: UPCR: Urine protein/creatinine ratio; MA: Microalbumin; A1M: α-1 microglobulin; S.G.: Specific gravity; Data presented as count, median (interquartile range, IQR), or mean ± standard deviation (SD); Bold indicates *p* < 0.05.

Notably, patient P9 in group A presented with compound heterozygous *ABCG8* mutations [c.490(exon4) C > T, c.323-1(IVS3) G > C], a specific causative gene of sitosterolemia, indicating the concurrent presence of two rare genetic disorders in this child.

### Analysis of genotype and phenotype

First, *NPHP1* mutations accounted for the largest proportion in both groups. Although all four patients with *NPHP1* variants in group B were female, this did not constitute a statistically significant gender difference compared to group A. In patients with *NPHP1* variants, the mean age at disease onset (13.7 ± 1.4 years) and progression to ESRD (13.7 ± 1.4 years) in group B were significantly higher than those in group A (9.1 ± 3.3 years and 10.5 ± 2.5 years, respectively; *p* < 0.05). In addition, group B exhibited more severe proteinuria than group A, with significantly higher UPCR (2.7 ± 1.3 vs. 0.6 ± 0.2 g/g, *p* < 0.001) and elevated median urinary MA/A1M ratios [3.6 (1.3–11.0) vs. 0.3 (0.1–0.6), *p* < 0.05] (Table 3).

**Table 3.** Phenotypic comparison of *NPHP1* variants in two groups.

Parameters	Group A (*n* = 9)	Group B (*n* = 4)	Total (*n* = 13)	*p*
Gender/male, *n* (%)	4 (44.4)	0 (0)	4 (30.8)	0.228
Age at onset (yr), mean ± SD	9.1 ± 3.3	13.7 ± 1.4	10.5 ± 3.5	**0.023**
Age at ESRD (yr), mean ± SD	10.5 ± 2.5^a^	13.7 ± 1.4	11.5 ± 2.6^b^	**0.042**
UPCR (g/g), mean ± SD	0.6 ± 0.2	2.7 ± 1.3	1.3 ± 1.2	**<0.001**
Urine MA /A1M, median (IQR)	0.3 (0.1–0.6)	3.6 (1.3–11.0)	0.4 (0.2–2.9)	**0.014**

Note: ESRD: End-stage renal disease; UPCR: Urine protein/creatinine ratio; MA: Microalbumin; A1M: α-1 microglobulin; Data presented as count, median (interquartile range, IQR), or mean ± standard deviation (SD); One patient in group A was currently in CKD4, so ^a^*n* = 8, ^b^*n* = 12; Bold indicates *p* < 0.05.

Among patients with non-*NPHP1* mutations (*n* = 8; group A:3, group B:5), 75% were male (Table 4). All four infantile-onset NPHP cases carried non-*NPHP1* mutations: *NPHP3* in group A, and *NPHP2*, *TTC21B* and *CCDC41* in group B. Except for one homozygous mutation (*NPHP3* p.L1073Ter,258; *TTC21B* p.R301C) and one single heterozygous mutation (*TTC21B* p.V626I; *CCDC41* p.L581P) per group, the remaining cases presented compound heterozygous mutations. Importantly, 80.0% (4/5) of group B patients with family history were non-*NPHP1* carriers.

**Table 4.** Phenotypic and genotypic comparison of non-*NPHP1* variants in two groups.

Group/Patient/gender	Mutation gene	Nucleotide change	Amino acid change	Zygosity	UPCR (g/g)	Age at onset (yr)	Age at ESRD (yr)	Extrarenal manifestations	Family history
A/P1/M	*NPHP3*	c.3757C > G c.875C > G∗	p.L1253V p.S292X∗	Het Het	0.63	1.25	11.58	–	–
A/P7/M	*NPHP3*	c.3218(exon23)T > G	p. L1073Ter,258	Hom	0.82	2.5	3	–	–
A/P6/M	*TTC21B*	c.1876G > A∗	p.V626I∗	Het	0.49	9.33	11.58	–	–
B/p1/F	*TTC21B*	c.1552T > C c.752T > G	p.C518R p.M251R	Het Het	3.22	1.25	5.58	HBP	Fetal death (elder siblings)
B/p2/M	*TTC21B*	c.901C > T	p.R301C	Hom	5.88	6.33	6.33	HBP, Situs inversus	First cousins (his parents)
B/p6/F	*TTC21B*	c.1552(exon13)T > C c.497(exon5) del A	p.C518R p.K166fs Ter36	Het Het	1.29	newborn	0.08	patent foramen ovale	Infant death (elder sister)
B/p4/M	*NPHP2*	c.1595(exon14) del G* c.1781-3(IVS14) c.1792 (exon 15) delAAGAAACAGCCAGAGinsTGGCT GTGAGGA*	p. S532fsTer39* -	Het Het	2.65	2.25	2.25	HBP	Uremia (uncle)
B/p7/M	*CCDC41*	c.1742(exon15) T > C*	p.L581P*	Het	1.23	1.42	3.25	–	–

Note: M: Male; F: Female; HBP: High blood pressure. *Novel mutation.

### *TTC21B* variants and proteinuria level

There was one patient with *TTC21B* single heterozygous mutation in group A and three patients with compound heterozygous or homozygous mutations in group B. Given that *TTC21B* has been identified as a causative gene for familial FSGS, we performed a literature review to investigate potential correlations between proteinuria severity and mutation zygosity (single heterozygous vs. biallelic) in *TTC21B*-associated nephropathy.

The systematic literature search was performed using the terms “TTC21B” OR “NPHP12” in PubMed, CNKI (China National Knowledge Infrastructure), and Wanfang Database, with a final search date of January 31, 2025. Studies were included if they: (1) reported clinical cases; (2) documented quantitative proteinuria measurements; and (3) featured moderate-to-heavy proteinuria (defined as urine protein ≥2+ by dipstick, or UPCR ≥1.0 g/g, or 24-h urine protein ≥1.0 g, or 24-h urine protein ≥25 mg/kg, or explicitly described as nephrotic-range proteinuria). We excluded mechanistic studies without clinical data and reports lacking proteinuria documentation.

This literature study enrolled 29 patients predominantly from North Africa, Europe, and China, stratified geographically into North Africa/Europe (*n* = 18) and Asia (*n* = 11) cohorts (Tables 5 and 6), with 12 males (Asia:5). The North Africa/Europe group demonstrated significantly later ESRD progression (17 years, IQR 7.5–32.0) compared to the Asian group (3.9 years, IQR 1.0–6.3; *p* = 0.001), excluding two non-ESRD cases.

**Table 5.** The *TTC21B* patients with moderate-to-heavy proteinuria and their phenotypes in literatures.

Country	Gender	Age at ESRD	Proteinuria (g/d)/renal biopsy	Extrarenal manifestations	*TTC21B* mutation	Reference
Tunisia	F	32	1.5, FSGS	HBP, myopia	p.P209L (Hom)	Huynh Cong et al. (2014) [31]
Tunisia	F	16	3.0, FSGS	HBP, cerebral aneurysm		
Algeria	M	22	2.5, ND	HBP		
Tunisia	M	27	2.9, FSGS	HBP		
Tunisia	F	35	7.0, MCNS	HBP		
Tunisia	F	17	Nephrotic-range, FSGS	NA		
Algeria	M	26, CKD4	1.0, FSGS	HBP		
Algeria	F	34	2.2, FSGS	Primary biliary cirrhosis		
Morocco	M	32	1.5, ND	HBP		
Spain	F	6	Nephrotic-range, FSGS	Myopia	p.P209L (Het) p.H426D (Het)	Bullich et al. (2017) [8]
Spain	M	8	Nephrotic-range, FSGS	Myopia		
Morocco	F	14	Nephrotic-range, Global sclerosis	HBP	p.P209L (Hom)	
Morocco	M	8	Nephrotic-range, FSGS	HBP, myopia		
Morocco	F	17 (not ESRD)	Nephrotic-range, FSGS	Myopia		
China	M	8	Proteinuria(78mg/kg/d), FSGS	HBP, situs inversus, short phalanges, growth retardation, neutropenia	c.2211 + 3A > G (Hom)	Zhang et al. (2018) [9]
China	F	1	Proteinuria (158mg/kg/ d), FSGS	HBP, hepatic fibrosis	p.C518R (Het) p.R486KfsX22(Het)	
China	F	4	0.556 (43 mg/kg/d), ND	HBP, situs inversus, short phalanges	p.C518R (Het) p.M251R (Het)	Jian et al. (2019) [41]
Japan	M	8	Proteinuria (33.8mg/kg/d), FSGS	Small liver cysts, dilated bile ducts	p.Y562C (Het) p.A857T (Het)	Hibino et al. (2020) [42]
Egypt	M	1.5	nephrotic-range, FSGS	HBP, right moderate hydronephrosis	p.P209L (Het) p.W150Ter (Het)	Abo El Fotoh and Al-Fiky. (2020) [43]
Czechia	F	2.5	nephrotic-range, FSGS	HBP, brachydactyly, disproportional growth, coned epiphyses of fingers	p.P209L (Het) p.C14R (Het)	Bezdíčka et al. (2021) [44]
North African	F	47	Proteinuria (UPCR 1.5), mild interstitial fibrosis, tubular atrophy	HBP, pre-eclampsia, myopia, cirrhosis	p.P209L (Hom)	Gambino et al. (2021) [45]
China	F	3.2	Proteinuria (++), ND	HBP, situs inversus	p.C518R (Het) p.R411X (Het)	Jing et al. (2022) [32]
China	M	3.9	Proteinuria (+++), FSGS	HBP, dextrocardia	p.C518R (Het) c.1675-1G > T (Het)	
	F	0.5	Proteinuria (++), ND	–		
China	M	2.2	Proteinuria (++), ND	HBP, growth retardation	p.C518R (Het) p.D177fs (Het)	
UK	F	3.6	Proteinuria (UPCR 2.1), FSGS	HBP, growth retardation	p.P209L (Het) p.Q834Ter (Het)	Olinger et al. (2022) [30]
China	F	5.58	Proteinuria (UPCR 3.22), FSGS	HBP	p.C518R (Het) p.M251R (Het)	Our study
	M	6.33	nephrotic-range, SGN	HBP, situs inversus	p.R301C (Hom)	
	F	0.08	Proteinuria (UPCR 1.29), ND	PFO	p.C518R (Het) p.K166fs Ter36 (Het)	

Note: ESRD: End-stage renal disease; M: Male; F: Female; ND: Not done; NA: Not available; UPCR: Urine protein/creatinine ratio; FSGS: Focal segmental glomerulosclerosis; MCNS: Minimal change nephrotic syndrome; SGN: Sclerosing glomerulonephritis; HBP: High blood pressure; PFO: Patent foramen ovale.

**Table 6.** Phenotypic and genotypic comparison of *TTC21B* variants in North Africa/Europe and Asia according to the literature.

Parameters	North Africa/Europe (*n* = 18)	Asia (*n* = 11)	Total (*n* = 29)	*P*
Gender /male, *n* (%)	7 (58.3)	5 (41.7)	12 (41.4)	0.446
Age at ESRD (yr), median (IQR)	17 (7.5-32)	3.9 (1-6.3)	8 (3.4-24)	**0.001**
Severe proteinuria, *n* (%)	10 (55.6)	5 (45.5)	15 (51.7)	0.853
FSGS, *n* (%)	13 (81.3)^a^	5 (83.3)^b^	18 (81.8)^c^	**0.003**
HBP, *n* (%)	13 (76.5)^d^	8 (72.7)	21 (75)^e^	>0.999
Myopia, *n* (%)	6 (35.3)^d^	0 (0)	6 (21.4)^e^	0.055
Situs inversus, *n* (%)	0 (0)^d^	4 (36.3)	4 (14.3)^e^	**0.016**
P209L variant, *n* (%)	18 (100)	0 (0)	18 (62.1)	**<0.001**
C518R variant, *n* (%)	0 (0)	8 (72.7)	8 (27.6)	**<0.001**

Note: ESRD: End-stage renal disease; FSGS: Focal segmental glomerulosclerosis; HBP: High blood pressure; Data presented as count or median (interquartile range, IQR); ^a^*n* = 16, ^b^*n* = 6, ^c^*n* = 22; The extrarenal manifestations of one patient in North African/Europe group was not available, so ^d^*n* = 17, ^e^*n* = 28; bold indicates *p* < 0.05.

We quantified the prevalence of severe proteinuria (defined as ≥3+ by dipstick, or UPCR ≥2.0 g/g, or 24-h urine protein >3.5 g, or 24-h urine protein ≥50 mg/kg, or explicitly designated as nephrotic-range proteinuria [14,15]) in both cohorts, with no statistically significant differences observed. Renal biopsies (22/29 patients, 75.9%) revealed FSGS in 81.8% of cases (18/22). While FSGS incidence was higher in the Asia group (*p* = 0.003), the lower biopsy rate in this group may introduce selection bias.

Three quarters of patients had presented hypertension, with no statistical intergroup difference. Distinct extrarenal manifestations emerged between groups: 6 North African/European patients exhibited myopia, while 4 Asian patients presented with situs inversus. All patients harbored biallelic (compound heterozygous/homozygous) mutations. The P209L variant (homozygous/heterozygous) was ubiquitous in North Africa/Europe patients, whereas the C518R heterozygous mutation showed exclusive Asian predominance (72.7 vs. 0%, *p* < 0.001).

## Discussion

NPHP, a ciliopathy-mediated tubulointerstitial nephropathy, poses diagnostic challenges due to nonspecific early symptoms (fatigue/anemia in most cases). While previous reports [4,7,16] showed that about 50% of patients had extrarenal manifestation (predominantly retinopathies), none of the patients in our cohort presented with retinopathy, and only a few had other extrarenal manifestations. The median age at progression to ESRD in this study was younger, which may be due to the exclusive inclusion of pediatric patients (≤18 years). Compared with some other studies [5,16], the prevalence of hyposthenuria in our study was much lower. Whether these differences are related to regional variations or the limited sample size remains to be further explored.

Renal ultrasonography identified kidney enlargement in only three children (group A:2 [*NPHP1*, *NPHP3*]; group B:1 [*NPHP2*]), notably including patient P1 (*NPHP3*) who presented with childhood-onset microalbuminuria and nephromegaly progressing to renal atrophy and ESRD over 10 years, consistent with literature reports of *NPHP3′*s association with renal enlargement [10,17,18]. The *NPHP3* gene (3q22.1) encodes nephrocystin-3, a proximal primary cilium protein that interacts with inversin (INVS). Through ciliary structural disruption, this protein complex induces multiorgan cyst formation [19,20]. Given its status as a potential second most prevalent NPHP gene in China [16] and a major cause of infantile NPHP [21], *NPHP3* should be prioritized in pediatric cases exhibiting low-molecular-weight proteinuria with nephromegaly.

In this study, no significant differences were observed in gender distribution, age of onset, age at progression to ESRD, or growth retardation between the two groups. Notably, the incidence of edema and hypertension in group B was significantly higher than that in group A. These manifestations, being associated with glomerular dysfunction, suggest potential glomerular involvement in NPHP pathogenesis. Moreover, group B showed significantly higher urinary albumin levels and MA/A1M ratios, further supporting the hypothesis of glomerular-origin proteinuria in these patients.

Since NPHP is an autosomal recessive disease lacking specific renal pathological features [22–24], we focused on genetic analysis. *NPHP1* is the most frequently mutated gene in NPHP (>50% cases), which is consistent with Petzold’s landmark cohort study [7]. Nephrocystin-1, encoded by the *NPHP1* gene, is an adaptor protein localized to both primary cilia and apical surface of renal epithelial cells. It plays essential roles in cell adhesion, signaling, and maintenance by interacting with tyrosine kinase-2, tensin [25], and other renal cysteines [26]. Patients with NPHP1 variants typically present with mild low-molecular-weight proteinuria and progression to ESRD, while moderate proteinuria is rarely observed (Supplementary Figure 2). At present, there are no reports about *NPHP1* acting on glomeruli, but improved glomerulosclerosis has been observed in Nphp1^del2-20/del^ [2–20] knockout mice with re-expression of Nephrocystin-1 [27]. While all *NPHP1* variants were large deletions in both groups, group B exhibited significantly more severe proteinuria. This was particularly evident in patient p5, who presented with nephrotic-range proteinuria (the genetic testing results for patient p5 and her family are presented in Supplementary Figure 3). Unexpectedly, both the disease onset and progression to ESRD of *NPHP1* patients in group B occurred significantly later. Therefore, further in-depth research is needed to explore whether glomerular injury is secondary to tubulointerstitial injury or if *NPHP1* directly acts on the glomerulus.

*TTC21B* (NPHP12) emerged as group B’s second most frequent mutation, encoding IFT139 as a core IFT-A complex component critical for ciliary retrograde transport [28,29]. Pathogenic variants of *TTC21B* account for approximately 5% of ciliopathy case [28]. In Petzold’s NPHP cohort [7], the incidence of *TTC21B* mutations was 6%, second only to *NPHP1*. Patients with *TTC21B* mutations frequently present with extrarenal manifestations, including myopia, situs inversus, cerebral aneurysm, hypertension and so on [9,30]. Cong et al. [31] identified *TTC21B* as a causative gene for familial FSGS. They proposed that this effect was potentially mediated through IFT139’s cilia-independent regulation of podocyte cytoskeletal organization, thus explaining severe proteinuria/edema and FSGS pathology [9,32]. Consistent with previous reports, three patients (75%) with *TTC21B* mutations in this cohort demonstrated moderate-to-heavy proteinuria accompanied with hypertension and edema. One patient’s early-stage renal biopsy revealed FSGS pathology, further supporting *TTC21B* as an established causative gene for hereditary FSGS.

As of March 18, 2025, HGMD documents 122 *TTC21B* mutations (58.2% missense), with P209L prevalent in North Africa and Portugal [9,31], and C518R in Chinese populations [33]. These conclusions were confirmed by the literature review of this study where C518R heterozygotes (*n* = 2) showed moderate proteinuria and early ESRD. Previous studies showed that P209L homozygotes typically exhibit milder disease (late-onset ESRD). According to the literature review here, compound heterozygous P209L variants accelerate ESRD onset compared to homozygous cases. Studies suggest that *TTC21B* exhibits allelic heterogeneity, with compound heterozygous variants often resulting in more severe phenotypes than homozygous variants [28]. However, our R301C homozygote presented nephrotic-range proteinuria, hypertension, situs inversus, and ESRD at 6.33 years. Notably, this mutation has only been documented in a screening without any functional validation [34], highlighting the need for further investigation into *TTC21B*’s pathogenic mechanisms.

Although research on NPHP has been ongoing, approximately 30% of clinical NPHP cases failed to obtain molecular diagnosis [7]. While 27 NPHP-related genes have been identified to date, nearly two-thirds of NPHP-associated genes have remained unidentified [11,23,35]. In this study, we found five novel NPHP gene variants, expanding the known NPHP gene variation spectrum. Among them, patients P6 (*TTC21B*) and p7 (*CCDC41*) carried single heterozygous variants classified as variants of uncertain significance (VUS) per ACMG/AMP guidelines. *CCDC41* (NPHP18/CEP83) is known to cause infantile NPHP *via* IFT20 interaction [36,37], but documented cases remain scarce. Previous studies have indicated that *CCDC41* variants existed exclusively as either compound heterozygous or homozygous mutations [10,36,38]. However, single heterozygous mutations in ciliopathy genes have been frequently reported, possibly due to undetected second-allele variants by sequencing methods [4,39,40]. In addition, both presented with classic NPHP features: insidious onset, low-molecular-weight proteinuria, normal kidney size/blood pressure (p7 had bilateral cysts), and progression to ESRD. Given these considerations, we ultimately included both patients.

In conclusion, this study is the first to analyze NPHP patient phenotypes and genotypes based on proteinuria levels. Compared to the mild proteinuria group, patients with moderate-to-heavy proteinuria exhibited higher rates of family history, hypertension, and edema. We identified five novel NPHP gene mutations, broadening the disease’s molecular spectrum. Phenotype-genotype correlations showed that *NPHP3* patients often present with enlarged kidneys, while *TTC21B* emerged as a major pathogenic gene in cases with heavier proteinuria. However, this single-center study had a limited sample size, restricting the robustness of correlation analyses. Key unresolved questions include the link between moderate-to-heavy proteinuria and family history prevalence, the mechanism of phenotypic variability in *NPHP1* large exonic deletions, and the pathogenesis of renal enlargement in *NPHP3*. Future studies will expand the cohort to address these gaps.

## Supplementary Material

Supplementary Figure 1.tif

Supplementary table.docx

Suppl. Figure_3.tif

Supplementary Figure 2.tif

Supplementary figure legends.docx

## Data Availability

The data presented in this study are available on request from the corresponding author due to privacy and ethical considerations.
