# Supplementary figures and images for: Phenotypic and genotypic analysis of pediatric nephronophthisis patients with different levels of proteinuria
Source: Ren Fail. 2025 Dec 15;47(1):2598179. doi: 10.1080/0886022X.2025.2598179 (PMC12710272; doi:10.1080/0886022X.2025.2598179)

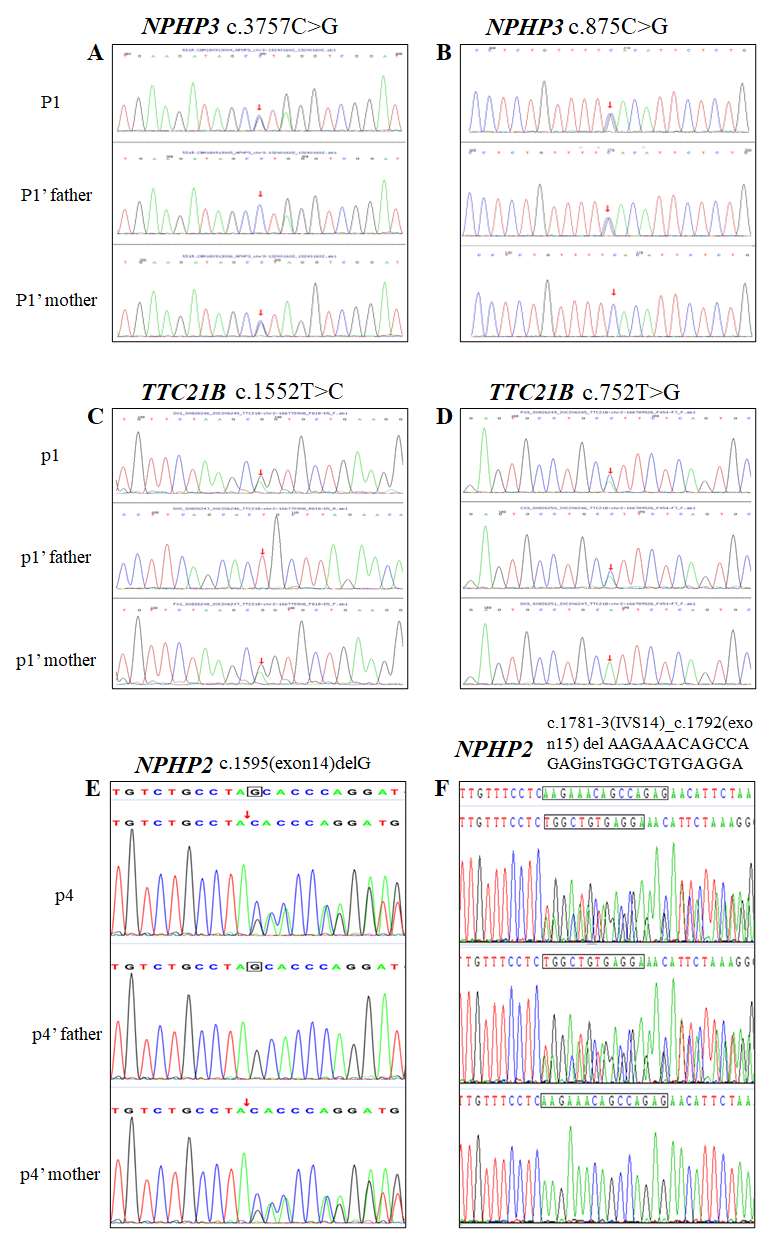

Supplement: Supplementary Figure 1.tif [file IRNF_A_2598179_SM8296.tif]

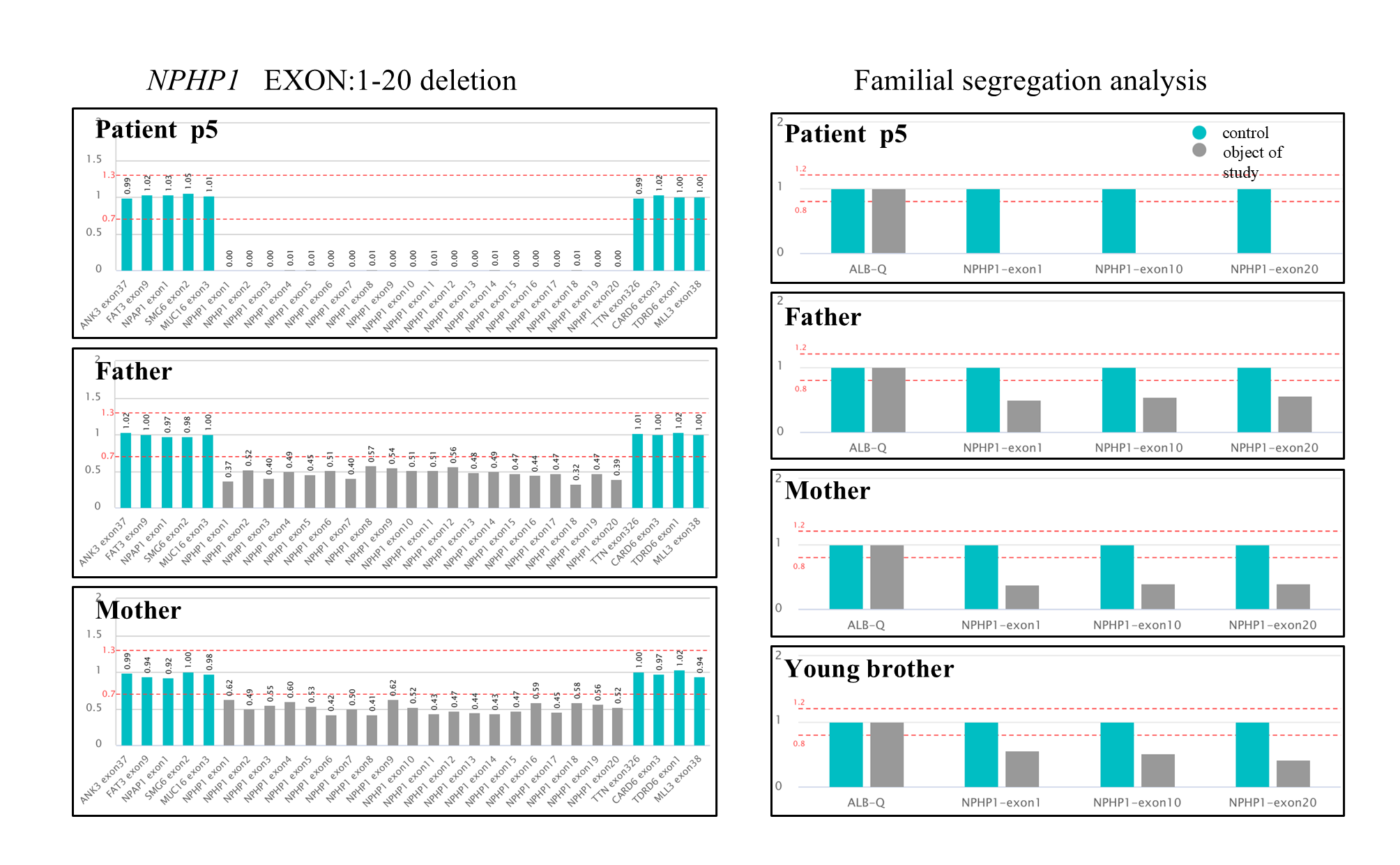

Supplement: Suppl. Figure_3.tif [file IRNF_A_2598179_SM8294.tif]

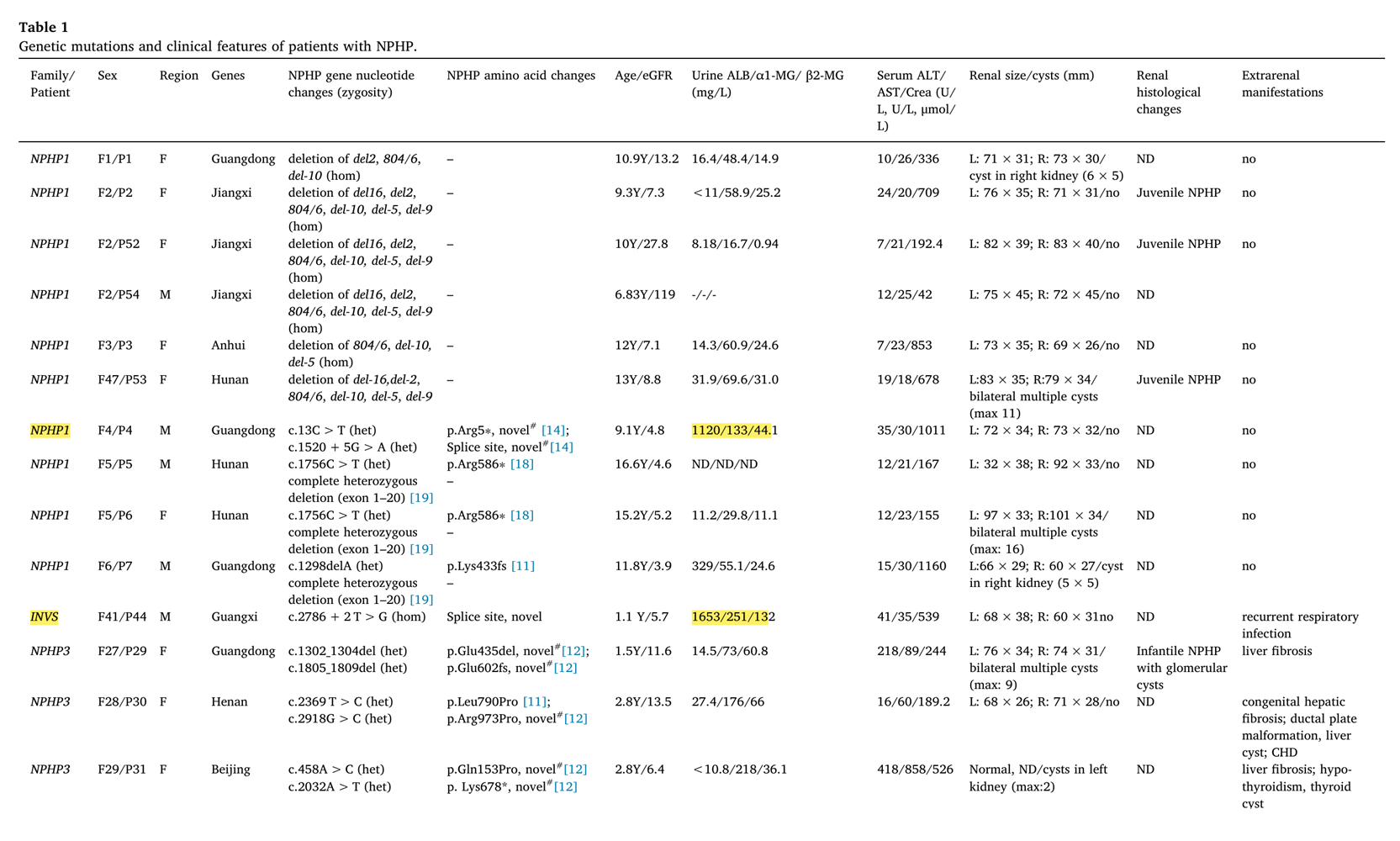

Supplement: Supplementary Figure 2.tif [file IRNF_A_2598179_SM8293.tif]
