# Supplementary material for: Phenotypic and genotypic analysis of pediatric nephronophthisis patients with different levels of proteinuria
Source: Ren Fail. 2025 Dec 15;47(1):2598179. doi: 10.1080/0886022X.2025.2598179 (PMC12710272; doi:10.1080/0886022X.2025.2598179)
Supplement: Supplementary table.docx [file IRNF_A_2598179_SM8295.docx]

**Supplementary Table 1.** Clinical data of two groups

| Patient/Gender | Age at onset (yr) | Symptoms of onset | With ESRD | Age at ESRD (yr) | Blood pressure | Urine output | Edema | Extrarenal manifestations | Family history |
| --- | --- | --- | --- | --- | --- | --- | --- | --- | --- |
| Group A (mild proteinuria) | | | | | | | | | |
| P1/M | 1.25 | Proteinuria by PE | Yes | 11.58 | norm | norm | - | - | - |
| **P2/F** | 7 | Anemia | Yes | 9.5 | norm | norm | - | - | - |
| **P3/F** | 13.67 | Anemia | Yes | 13.67 | norm | norm | - | - | - |
| **P4/F** | 8.75 | Anemia, Pain in limbs | Yes | 9.75 | norm | Polyuria | - | Polydipsia | - |
| **P5/M** | 7.42 | Anemia, Fatigue | Yes | 8.42 | norm | norm | - | - | - |
| P6/M | 9.33 | Anemia | Yes | 11.58 | norm | norm | - | - | - |
| P7/M | 2.5 | Anemia | Yes | 3 | norm | norm | - | - | - |
| **P8/M** | 5.5 | Anemia, nocturnal polyuria | No | - | norm | Polyuria | - | - | MN (grandmother) |
| **P9/M** | 10.42 | Anemia | Yes | 11.58 | norm | norm | - | thrombocytopenia, hypertriglyceridemiaxanthomatosis | - |
| **P10/F** | 5 | Anemia | Yes | 7 | norm | norm | - | cerebral palsy | - |
| **P11/F** | 14.17 | IAP | Yes | 14.17 | norm | norm | - | - | - |
| **P12/M** | 9.75 | Anemia | Yes | 9.75 | norm | norm | - | - | - |
| Group B (moderate-to-heavy proteinuria) | | | | | | | | | |
| p1/F | 1.25 | Proteinuria by PE | Yes | 5.58 | HBP | norm | + | - | Fetal death (elder siblings) |
| p2/M | 6.33 | Vomiting, edema | Yes | 6.33 | HBP | oliguria | + | situs inversus | First cousins (his parents) |
| **p3/F** | 13.42 | Anemia, LHF | Yes | 13.42 | HBP | oliguria | + | - | Uremia (grandmother) |
| p4/M | 2.25 | Anemia, Fatigue | Yes | 2.25 | HBP | norm | - | - | Uremia (uncle) |
| **p5/F** | 13.08 | Edema | Yes | 13.08 | HBP | decrease | + | - | - |
| p6/F | newborn | High Scr by PE | Yes | 0.08 | norm | norm | - | PFO | Infant death (elder sister) |
| p7/M | 1.42 | High Scr by PE | Yes | 3.25 | norm | norm | - | - | - |
| **p8/F** | 15.67 | Growth retardation | Yes | 15.67 | norm | norm | - | - | - |
| **p9/F** | 12.42 | Anemia, Fatigue | Yes | 12.42 | norm | norm | - | - | - |

Note: PE: physical examination; MN: Membranous nephropathy; Scr: serum creatinine; PFO: patent foramen ovale; IAP: Intermittent abdominal pain; HBP: high blood pressure; LHF: left heart failure.

**Supplementary Table 2.** Laboratory and imaging examination of two groups

| Patient | UPCR (mg/mg) | Urine MA/IGU/A1M (mg/L) | Renal size (mm) | Renal cysts by ultrasound | Renal histological changes | Causal gene | Gene mutation (zygosity) |
| --- | --- | --- | --- | --- | --- | --- | --- |
| Group A (mild proteinuria) | | | | | | | |
| P1 | 0.63 | 35.10/9.59/160 | L:81×31; R:81×32 (S) | NF | ND | *NPHP3* | c.3757C>G, p.L1253V(het)  c.875C>G, p.S292X (het) |
| **P2** | 0.59 | 17.10/5.21/66.1 | L:79×31; R:77×29 (S) | NF | ND | ***NPHP1*** | large deletions (hom): chr2:110881367-110962545 (*NPHP1, MALL*) |
| **P3** | 0.53 | 31.10/9.32/83.1 | L:81×38; R:77×36 (S) | In LK (28×22) | ND | ***NPHP1*** | large deletions (hom):  chr2:110873264-110962545 (*NPHP1, MALL*) |
| **P4** | 0.65 | 160/20.6/60.9 | L:76×31; R:77×30 (S) | Bilateral multiple cysts (max 12×7) | ND | ***NPHP1*** | large deletions (hom):  chr2:110881024-110980431 (NPHP1, LINC00116) |
| **P5** | 0.46 | 10.70/3.35/50.1 | L:87×40; R:83×39 (N) | NF | SGN, diffuse fibrosis of renal interstitium (80% -90%) | ***NPHP1*** | large deletions (hom):  chr2:110881342-110962608 (*NPHP1*) |
| P6 | 0.49 | 19.3/6.58/55.8 | L:88×36; R:82×31 (N) | NF | ND | *TTC21B* | c.1876G>A, p.V626I (het) |
| P7 | 0.82 | 52.6/3.12/61.6 | L:81×39; R:81×36 (L) | NF | Shrinkage of glomerular capillary loops, 70% renal interstitium with chronic lesions, and cystic dilation of renal tubules | *NPHP3* | c.3218(exon23)T>G， p.Leu1073Ter,258 (hom) |
| **P8** | 0.55 | 6.47/3.86/40.4 | L:78×35; R:76×32 (N) | NF | ND | ***NPHP1*** | large deletions (hom):  chr2:110881342-110970348 (*NPHP1*) |
| **P9** | 0.55 | 2.19/<3.00/55.6 | L:81×38; R:78×35 (S) | NF | Renal tubulointerstitial nephropathy with ischemic kidney damage | ***NPHP1***  *ABCG8* | large deletions (hom):  chr2:110881367-110962545 (*NPHP1, MALL*) |
| **P10** | 0.44 | 18.2/8.06/42.1 | L:81×30; R:81×30 (N) | NF | ND | ***NPHP1*** | large deletions (hom):  chr2:110880924-110962590 (*NPHP1, MALL*) |
| **P11** | 0.89 | 87.8/18/113 | L:78×34; R:79×26 (S) | multiple cysts in RK (max 13×9) | ND | ***NPHP1*** | large deletions (hom):  chr2:110881367-110962545 (*NPHP1, MALL*) |
| **P12** | 0.78 | 10.4/<3.00/145 | L:90×39; R:91×41 (L) | NF | ND | ***NPHP1*** | large deletions (hom):  chr2:110873242-110970348 (*NPHP1*) |
| Group B (moderate-to-heavy proteinuria) | | | | | | | |
| p1 | 3.22 | 1150/8.83/10.05 | L:70×29; R:69×30 (N) | NF | FSGS | *TTC21B* | c.1552T>C, p.C518R (het); c.752T>G, p.M251R (het) |
| p2 | 5.88 | 4280/147/321 | L:68×29; R:69×30 (S) | NF | SGN, diffuse fibrosis of renal interstitium (90%) | *TTC21B* | c.901C>T, p.R301C (hom) |
| **p3** | 3.41 | 404/66.4/560 | L:71×28; R:68×28 (S) | NF | ND | ***NPHP1*** | large deletions (hom):  chr2:110881348-110962565 (*NPHP1*) |
| p4 | 2.65 | 860/26.2/144 | L:75×30; R:73×29 (L) | Bilateral multiple cysts (max 8×6) | ND | *NPHP2* | c.1595(exon14) delG, p.Ser532fsTer39 (het);  c.1781-3 (IVS14)_c.1792 (exon15)delAAGAAACAGCCAGAGinsTGGCTGTGAGGA (het) |
| **p5** | 4.14 | 1060/106/79.5 | L:83×37; R:82×36 (S) | NF | ND | ***NPHP1*** | large deletions (hom):  chr2:110881367-110962545 (*NPHP1, MALL*) |
| p6 | 1.29 | 185/12.6/135 | L:33×15; R:32×13 (S) | NF | ND | *TTC21B* | c.1552(exon13) T>C, p.C518R (het);  c.497(exon5) del A, p.Lys166fs Ter36 (het) |
| p7 | 1.23 | 208/5.27/105 | L:50×29; R:63×30 (N) | Bilateral multiple cysts (max 6×5) | ND | *CCDC41* | c.1742(exon15) T>C, p.L581P (het) |
| **p8** | 1.59 | 308/29/99.7 | L:75×32; R:72×31 (S) | NF | ND | ***NPHP1*** | large deletions (hom):  chr2:110881348-110962565 (*NPHP1*) |
| **p9** | 1.72 | 345/45.5/83.4 | L:79×32; R:79×32 (S) | Bilateral multiple cysts (max 15×14) | ND | ***NPHP1*** | large deletions (hom):  chr2:110881367-110962545 (*NPHP1, MALL*) |

Note: UPCR: Urine protein/creatinine ratio; MA: microalbumin; IGU: urinary immunoglobulin G; A1M:α-1 microglobulin; S: small; N: normal; L: large; NF: not found; ND: not done; LK: left kidney; SGN: sclerosing glomerulonephritis; FSGS: focal segmental glomerulosclerosis; Normal reference range: MA<19mg/L, IGU<8mg/L, A1M<12.5mg/L.

**Supplementary Table 3.** Laboratory and imaging examination of two groups

| Patient/Gende | Age of first visit to our center (yr) | Weight(kg) | Height(mm) | BMI (kg/m^2^) | Hematuria | Urine glucose | Urine S.G. | Urine  MA/A1M | Renal transplant |
| --- | --- | --- | --- | --- | --- | --- | --- | --- | --- |
| Group A (mild proteinuria) | | | | | | | | | |
| P1/M | 11.58 | 37 | 147(<P50) | 17.12 | (-) | (±) | 1.005 | 0.22 | Yes |
| **P2/F** | 9.5 | 24.5 | 128(<P10) | 14.95 | (-) | (-) | 1.002 | 0.26 | Yes |
| **P3/F** | 13.67 | 31 | 151(<P25) | 13.60 | (-) | (±) | 1.002 | 0.37 | Yes |
| **P4/F** | 9.75 | 22 | 131(<P25) | 12.82 | (-) | (-) | 1.002 | 2.63 | Yes |
| **P5/M** | 8.42 | 45 | 139(>P50) | 23.29 | (-) | (-) | 1.008 | 0.21 | Yes |
| P6/M | 9.58 | 27 | 137(P50) | 14.39 | (-) | (-) | 1.014 | 0.35 | Yes |
| P7/M | 3 | 13 | 95(<P50) | 14.40 | (-) | (-) | 1.004 | 0.85 | Yes |
| **P8/M** | 5.67 | 19 | 119(>P50) | 13.42 | (-) | (-) | 1.010 | 0.16 | No |
| **P9/M** | 10.42 | 33 | 133(<P10) | 18.66 | (-) | (-) | 1.008 | 0.04 | No |
| **P10/F** | 6.5 | 19 | 114(<P25) | 14.62 | (-) | (-) | 1.009 | 0.43 | No |
| **P11/F** | 14.17 | 34.8 | 147(<P3) | 16.10 | (-) | (-) | 1.003 | 0.78 | Yes |
| **P12/M** | 9.75 | 37.8 | 140(>P50) | 19.29 | (-) | (±) | 1.004 | 0.07 | Yes |
| Group B (moderate-to-heavy proteinuria) | | | | | | | | | |
| p1/F | 2.33 | NF | NF | NF | (-) | (-) | 1.010 | 114.43 | Yes |
| p2/M | 6.33 | 17.9 | 120(>P50) | 12.43 | (-) | (-) | 1.024 | 13.33 | No（PD） |
| **p3/F** | 13.42 | 28 | 146(<P10) | 13.14 | (-) | (±) | 1.013 | 0.72 | Yes |
| p4/M | 2.25 | 13.5 | 92(>P50) | 15.95 | (-) | (-) | 1.012 | 5.97 | Yes |
| **p5/F** | 13.08 | 42.5 | 157(>P50) | 17.24 | (-) | (-) | 1.007 | 13.33 | Yes |
| p6/F | 0.08 | 2.7 | NF | NF | (-) | (+) | 1.006 | 1.37 | die |
| p7/M | 1.42 | 9.5 | 78(P10) | 15.61 | (-) | (-) | 1.007 | 1.98 | CKD 5 |
| **p8/F** | 15.67 | 35.6 | 150(<P3) | 15.82 | (-) | (±) | 1.005 | 3.09 | Yes |
| **p9/F** | 12.42 | 29.5 | 107(<P3) | 25.77 | (-) | (-) | 1.004 | 4.14 | Yes |

Note: S.G.: specific gravity, normal reference range 1.003~ 1.03; CKD: chronic kidney disease; PD: peritoneal dialysis; MA: microalbumin; A1M: α-1 microglobulin; NF: not found.
