# Supplementary material for: Phenotypic and genotypic analysis of pediatric nephronophthisis patients with different levels of proteinuria
Source: Ren Fail. 2025 Dec 15;47(1):2598179. doi: 10.1080/0886022X.2025.2598179 (PMC12710272; doi:10.1080/0886022X.2025.2598179)
Supplement: Supplementary figure legends.docx [file IRNF_A_2598179_SM8292.docx]

**Figure Legends**

**Supplementary Figure 1. Verification results of patients with compound heterozygous mutations.** The WES results of three patients (P1 in group A, p1/p4 in group B) revealed compound heterozygous mutations (P1: *NPHP3*; p1: *TTC21B*; p4: *NPHP2*). Parental segregation analysis by Sanger sequencing confirmed the variants were on different alleles.

**Supplementary Figure 2.** Our literature review identified rare cases with severe proteinuria. In the case series (Zhihui Yue., *Clin Chim Acta*, 2020,506:136-144.) presented in Figure 2, two highlighted patients (with *NPHP1* and *INVS* mutations respectively) both exhibited marked proteinuria (yellow highlight).

**Supplementary Figure 3.** Genetic testing of patient p5 (group B) presenting with severe proteinuria identified whole-exon *NPHP1* deletions, which were subsequently confirmed in both parents and one sibling.
